# Gut microbiome characteristics of women with hypothyroidism during early pregnancy detected by 16S rRNA amplicon sequencing and shotgun metagenomic

**DOI:** 10.3389/fcimb.2024.1369192

**Published:** 2024-08-09

**Authors:** Lin Hu, Yajuan Xu, Jingjing Li, Miao Zhang, Zongzong Sun, Yanjie Ban, Xin Tian, Dong Liu, Lulu Hu

**Affiliations:** Department of Obstetrics and Gynecology, The Third Affiliated Hospital of Zhengzhou University, Zhengzhou, China

**Keywords:** hypothyroidism, pregnancy, gut microbiome, 16S rRNA amplicon sequencing, shotgun metagenomic sequencing

## Abstract

**Objective:**

This study aimed to explore the correlation between microbiota dysbiosis and hypothyroidism in early pregnancy by 16S rRNA amplicon sequencing combined with metagenomic sequencing.

**Methods:**

Sixty pregnant women (30 with hypothyroidism and 30 normal controls) were recruited for 16S rRNA amplicon sequencing, and 6 patients from each group were randomly selected for metagenomic sequencing to assess the gut microbiome profile.

**Results:**

The 16S rRNA results showed that beta-diversity in the hypothyroidism group was decreased. The relative abundances of the *Prevotella* and *Paraprevotella* genera increased in the hypothyroidism group, and *Blautia* predominated in the controls. The metagenomics results revealed that *Prevotella_stercorea_CAG_629*, *Prevotella_hominis*, *Prevotella_sp_AM34_19LB*, etc. were enriched in the hypothyroidism group at the species level. Functional analysis revealed that the pyridoxal 5’-phosphate synthase pdxT subunit module was decreased, and the short-chain fatty acid (SCFA) transporter and phospholipase/carboxylesterase modules were strongly enriched in the hypothyroidism group. Hypothyroidism patients had increased C-reactive protein (CRP), interleukin-2 (IL-2), IL-4, IL-10, and tumor necrosis factor (TNF)-α levels. The pyridoxal 5’-phosphate synthase pdxT subunit, the SCFA transporter, and the phospholipase/carboxylesterase module were associated with different *Prevotella* species.

**Conclusion:**

In early pregnancy, women with hypothyroidism exhibit microbiota dysbiosis, and *Prevotella* may affect the metabolism of glutamate, SCFA, and phospholipases, which could be involved in the development of hypothyroidism during pregnancy.

## 1 Introduction

Hypothyroidism is a systemic hypometabolic syndrome caused by decreased synthesis and secretion of thyroid hormones or insufficient target tissue effect, with a 4% incidence (MacDonald and Monteleone, 2005; Dong and Stagnaro-Green, 2019). The demand for thyroid hormone by the mother and fetus increases during pregnancy, but the fetus cannot synthesize thyroid hormone until 20 weeks of gestation (Lee et al., 2020). Maternal hypothyroidism can increase the risk of miscarriage, premature birth, gestational hypertension, low birth weight infants, and neurodevelopmental delays in offspring (Alexander et al., 2017; Ge et al., 2020). However, the pathogenesis of hypothyroidism during pregnancy remains unclear.

The gut microbiome, as a current research hotspot, plays a crucial role in host immunity, endocrinology, metabolism, and other aspects. It may affect the intestinal barrier through “leaky gut” and “molecular mimicry” mechanisms and impact the host metabolism in inflammatory and autoimmune diseases (Benvenga and Guarneri, 2016; Thaiss et al., 2018). Wu et al. found that the abundance of *Prevotella* was increased in pregnant women with thyroid peroxidase antibody (TPOAb)-positive subclinical hypothyroidism in the second trimester (Wu et al., 2022). Therefore, microbiota dysbiosis is closely related to hypothyroidism during pregnancy. However, there are no comprehensive studies on the relationship between the characteristics of the gut microbiome and hypothyroidism in early pregnancy.

In this study, we applied 16S rRNA amplicon sequencing combined with shotgun metagenomic sequencing to investigate alterations of the gut microbiome in women with hypothyroidism in early pregnancy and explored the role of microbiota dysbiosis in the pathogenesis of hypothyroidism during pregnancy.

## 2 Materials and methods

### 2.1 Study subjects

Pregnant women who received perinatal care at the Third Affiliated Hospital of Zhengzhou University, China between November 2021 and May 2022 were recruited. Thirty pregnant women with hypothyroidism who satisfied the inclusion criteria were included in the hypothyroidism group, whereas thirty normal pregnant women were included in the control group during the same time period. From the hospital’s electronic medical records, we collected demographic details and relevant clinical data of the participants.

The inclusion criteria were as follows: (1) Thyroid function level complied with the diagnostic criteria of the Guideline on Diagnosis and Management of Thyroid Diseases during Pregnancy and Postpartum (2nd edition) (Ad Hoc Writing Committee for Guideline on Diagnosis and Management of Thyroid Diseases during Pregnancy and Postpartum et al., 2019) for hypothyroidism during pregnancy and the diagnostic criteria formulated by the Clinical Laboratory of the Third Affiliated Hospital of Zhengzhou University, China [serum thyroid stimulating hormone (TSH)>4.2 mIU/L] and (2) Gestational age less than 14 weeks.

The exclusion criteria were as follows: (1) age<18 years or ≥35 years; (2) multiple pregnancies; (3) artificial conception; (4) severe stress, anxiety, or depression; (5) endocrine or immune system diseases before and during pregnancy, such as diabetes, gestational hypertension and systemic lupus erythematosus; (6) severe gastrointestinal diseases or gastrointestinal surgery; and (7) received antibiotic, probiotic, or immunosuppressant treatment in the previous 2 months. and (8) applied anti-thyroid drugs or thyroid hormone replacement.

The study was approved by the Medical Ethics Committee of the Third Affiliated Hospital of Zhengzhou University, China (NO: 2021-105-01). All enrolled subjects participated voluntarily and signed an informed consent form.

### 2.2 Sample collection

All fecal samples were collected from all subjects within 24 hours of diagnosis of hypothyroidism during pregnancy. Before collecting the samples, the subjects were informed. After natural defecation, a sterile spoon was used to carefully collect the sample from the middle part of the stool, ensuring that the sample did not contact the bedpan to avoid mixing with urine. After collection, the sample was placed in a 2.0 mL sterile tube, transported to the laboratory within 2 hours, and stored at -80°C until further processing.

### 2.3 Laboratory testing

Laboratory testing was conducted in the laboratory department of the Third Affiliated Hospital of Zhengzhou University, China. All tests were performed by the manufacturer’s instructions.

The levels of serum thyroid-stimulating hormone (TSH), free thyroxine (FT4), TPOAb, and thyroglobulin antibody (TgAb) were measured using the Cobas e 801 electrochemiluminescence immunoassay analyzer (Roche, USA) and its supporting kit (Roche, Jiangxi, China). The normal reference values were established as follows: TSH (0.27-4.2 mIU/L), FT4 (12-22 pmol/L), TPOAb (0-34 IU/mL), and TgAb (0-114 IU/mL).

Serum fasting blood glucose (GLU), total cholesterol (TC), triglyceride (TG), low-density lipoprotein cholesterol (LDL-C) and high-density lipoprotein cholesterol (HDL-C) levels were measured by an AU5800 automatic biochemistry analyzer (Beckman Coulter, USA) and matching kit (Beckman Coulter, Suzhou, China). The normal reference values were established as follows: GLU (3.9-6.1 mmol/L), TC (0-6.2 mmol/L), TG (0.4-1.86 mmol/L), LDL-C (1.9-3.8 mmol/L), and HDL-C (1.29-1.55 mmol/L).

The levels of C-reactive protein (CRP) and hemoglobin (HGB) in peripheral blood were tested by the BC-5700 (Mindray, China) with a corresponding kit (Mindray, Shenzhen, China). The normal reference values were established as follows: CRP (0-3 mg/L) and HGB (115-150 g/L).

The levels of peripheral blood interleukin (IL)-2, 6, 8, 10 and tumor necrosis factor (TNF)-α were determined by a FACSCanto™ flow cytometer (BD, USA) and a human Th1/Th2 subset detection kit (Cellgene biotech, Jiangxi, China). The normal reference values were established as follows: IL-2 (≤ 11.4 pg/ml), IL-4 (≤ 12.9 pg/ml), IL-6 (≤ 20 pg/ml), IL-10 (≤ 5.9 pg/ml), and TNF-α (≤ 5.5 pg/ml).

### 2.4 16S rRNA amplicon sequencing and bioinformatics analysis

16S rRNA amplicon sequencing was used to analyze the fecal samples from all participants. A MagPure Stool DNA KF Kit B (cat. no.MD511, Magen, China) was used to extract total DNA from the microbial community in the stool specimens (0.20 g) (Fadrosh et al., 2014). Polymerase chain reaction (PCR) was used to amplify the highly variable V3-V4 region of the bacterial 16S rRNA gene. The PCR primer sequences were 338F (5’-ACTCCTACGGGAGGCAGCAG-3’) and 806R (5’-GGACTACHVGGGTWTCTAAT-3’). Magnetic beads were used for purification, and library construction was completed. Sequencing was performed using the Illumina MiSeq platform (BGI, Shenzhen, China) to generate 300 bp paired-end reads. After obtaining the Illumina raw data in fastq format, quality control and filtering were performed to obtain CleanData. The 60 samples generated 8,171,576 16S rRNA reads (mean reads per sample = 68,096). After sequence splicing, tags for the hypervariable region were obtained using the FLASH software (version 1.2.1, https://github.com/dstreett/FLASH2) (Magoc and Salzberg, 2011). The software USEARCH (version 7.0.1090, http://www.drive5.com/usearch/) (Edgar, 2013) was used to cluster tags according to 97% sequence similarity to generate operational taxonomic units (OTUs). The software RDP classifier (version 2.2, https://github.com/rdpstaff/classifier) (Wang and Cole, 2024) was used to compare representative OTUs sequences with the database for species annotation. The software Mothur (version 1.31.2, http://www.mothur.org) (Chappidi et al., 2019) was used for alpha diversity (Chao, ACE, Shannon and Simpson indices) analysis, and software QIIME(version 1.80, http://qiime.org/1.8.0/) (Hall and Beiko, 2018) was used for beta diversity analysis via principal coordinate analysis (PCoA) and nonmetric multidimensional scaling (NMDS). The Wilcoxon rank-sum test and linear discriminant analysis effect size (LEfSe) analysis were used to identify species with significant differences in microbiome abundance between the two groups.

### 2.5 Shotgun metagenomic sequencing and bioinformatics analysis

Fecal samples from six hypothyroid patients and six control group participants were chosen by simple random sampling and evaluated by metagenomics sequencing. A MagPure Stool DNA KF Kit B (cat. no.MD511, Magen, China) was used to extract total DNA from the microbial community in the stool specimens (0.20 g). The quality and quantity of the extracted DNA were determined using a microplate reader, and the DNA fragment size was assessed by agarose gel electrophoresis. One microgram of genomic DNA was used, and a Covaris instrument was used to ultrasonically interrupt it to obtain a 300 bp fragment. Fastp and the software MEGAHIT (version 1.1.2, https://github.com/voutcn/megahit) (Li et al., 2015) were used for quality control and assembly. The 12 samples generated 73,022,905,500 metagenomic reads, and the average assembly length was 123.16 M.

The software Prodigal (version 2.6.3, https://github.com/hyattpd/Prodigal) (Hyatt et al., 2010) was used for metagenomic gene prediction. The software Diamond (version 2.0.13, https://github.com/bbuchfink/diamond) (Gautam et al., 2022) was used to annotate the gene set in the Kyoto Encyclopedia of Genes and Genomes (KEGG) Database (https://www.genome.jp/kegg/) to obtain KEGG orthologous (KO) abundance tables. The software CD-HIT (version 4.8.1, https://github.com/weizhongli/cdhit) (Kondratenko et al., 2020) was used for clustering to obtain nonredundant gene sets, and the software BLAST (version 2.2.28, http://blast.ncbi.nlm.nih.gov/Blast.cgi) (Schmid et al., 2023) was subsequently used for comparison with the Non-Redundant Protein Sequence (NR) Database (https://ftp.ncbi.nlm.nih.gov/blast/db/FASTA/). The Wilcoxon rank-sum test was used to evaluate differences.

### 2.6 Statistical analysis

All statistical analyses of 16S rRNA amplicon sequencing and metagenomic sequencing data were performed in R (version 3.4.1). SPSS software (version 26.0, IBM, USA) was used for analysis. Normally distributed measurement data are described as the mean ± standard deviation (SD) according to the t test. Nonnormally distributed measurement data are expressed as medians and quartiles by Wilcoxon rank-sum tests. Categorical variables are reported as frequencies [n (%)] according to the Chi-square test. Correlations were examined by Spearman rank correlation analysis. All the statistical analyses were two-tailed tests, with p < 0.05 indicating statistical significance.

## 3 Results

### 3.1 General clinical data

Age differences, body mass index (BMI) at enrollment, gestational age, TPOAb, TgAb, TC, TG, LDL-C, HDL-C, GLU, and HGB were not significant between the hypothyroidism and control groups (P > 0.05).Higher serum CRP and lower FT4 levels were observed in the hypothyroidism group than in the control group (**Table 1**). The clinical characteristics of the 12 subjects who received shotgun metagenomics are displayed in **Supplementary Table 1** of the **Supplementary Materials**.

**Table 1:** Clinical characteristics of the participants.

Parameter	Hypothyroidism (n=30)	Control (n=30)	P value
Maternal age, yr*	29.63 ± 3.65	30.80 ± 2.55	0.135
BMI, kg/m^2^*	23.09 ± 2.88	22.92 ± 3.70	0.846
Gestational age, weeks*	9.57 ± 2.79	10.41 ± 2.74	0.240
TSH, mIU/L*	5.23 ± 1.49	1.67 ± 0.81	**<0.001**
FT4, mIU/L*	15.17 ± 2.58	16.9 ± 2.28	**0.007**
TgAb, n (%)	2 (6.7%)	0 (0)	0.472
TPOAb, n (%)	2 (6.7%)	1 (3.3%)	1.000
TG, mmol/L#	1.45 (1.06, 1.93)	1.28 (0.95, 1.69)	0.098
TC, mmol/L#	4.62 (1.02, 5.19)	4.28 (3.94, 5.27)	0.549
LDL-C, mmol/L#	2.90 (1.55, 2.05)	2.56 (2.43, 3.14)	0.318
HDL-C, mmol/L#	1.84 (2.50, 3.23)	1.89 (1.64, 2.02)	0.631
GLU, g/L*	4.82 ± 0.43	4.72 ± 0.51	0.415
HGB, g/L*	123.53 ± 9.97	126.33 ± 9.08	0.260

P<0.05 was significant. *Data are expressed as means ± standard deviation. #Data are expressed as median (P25, P75). BMI, body mass index; TSH, thyroid stimulating hormone; FT4, free T4; TgAb, anti-thyroglobulin antibodies; TPOAb, thyroid peroxidase antibody; TC, total cholesterol; TG, triglycerides; LDL-C, low-density lipoprotein cholesterol; HDL-C, high-density lipoprotein cholesterol; GLU, fasting blood glucose; and HGB, hemoglobin.Boldface indicates statistical significance.

### 3.2 Microbial taxa alteration determined by 16S rRNA amplicon sequencing

**Figure 1:**
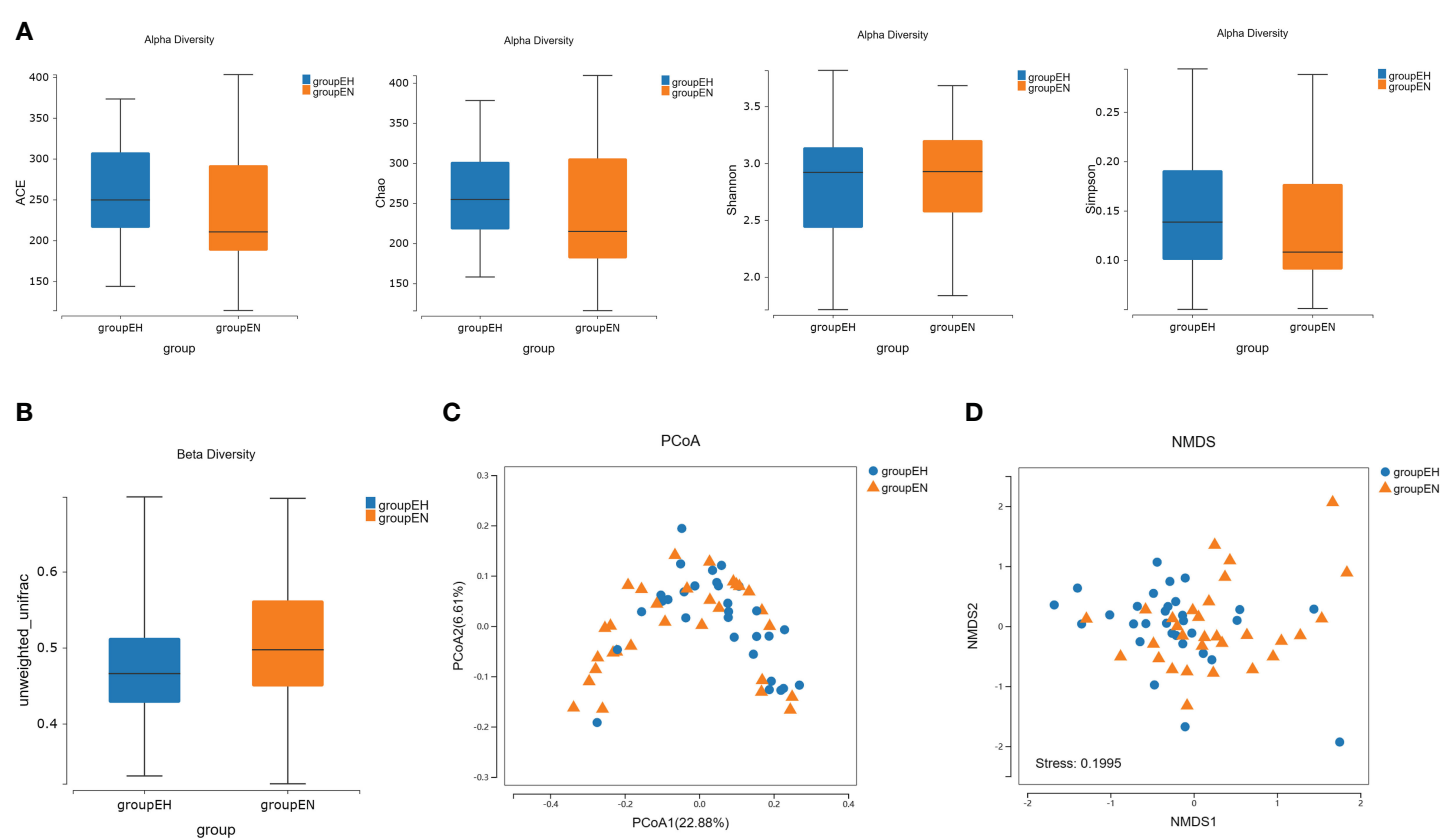
Taxonomic diversity and composition analysis by 16S rRNA sequencing. **(A)** The alpha diversity analysis (ACE index, P = 0.060; Chao index, P = 0.060; Shannon index, P = 0.582; Simpson index, P = 0.374) of the two groups by the Wilcoxon rank-sum test. **(B)** The beta diversity analysis based on unweighted-UniFrac distance, P<0.001. **(C)** PCoA analysis in two groups. P = 0.014, PC1 explained 22.88% of the variation, and PC2 explained 6.61% of the variation. **(D)** NMDS analysis in two groups, stress = 0.200, and ANOSIM test, R = 0.067, P = 0.004. groupEH, the hypothyroidism group; groupEN, the control group.

There was no significant difference in the alpha diversity between the hypothyroidism group and the control group (**Figure 1A**). Based on the unweighted UniFrac distance, beta diversity was significantly different between the two groups (**Figure 1B**). PCoA showed that the difference between the two groups was statistically significant (**Figure 1C**). A segregation trend between the two groups was observed by NMDS analysis (**Figure 1D**).

**Figure 2:**
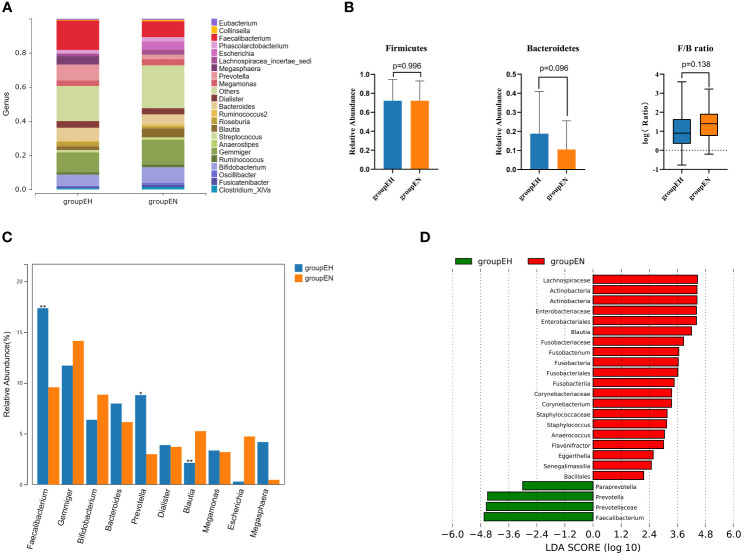
Taxonomic composition analysis by 16S rRNA sequencing. **(A)** The gut microbiome composition diagrams at the genus level. The species whose abundance were less than 0.5% were classified into others. **(B)** Firmicutes/Bacteroidetes ratio in two groups. **(C)** The top 10 genera were selected for comparison. **(D)** LEfSe analysis in two groups, species with P<0.05 and linear discriminant analysis (LDA) scores > 2 were displayed. groupEH, the hypothyroidism group; groupEN, the control group. *P<0.05, **P<0.01.

At the genus level (**Figure 2A**), the top 5 dominant bacterial genera were *Faecalibacterium*, *Gemmiger*, *Prevotella*, *Bacteroides* and *Bifidobacterium* in the hypothyroidism group and *Gemmiger*, *Faecalibacterium*, *Bifidobacterium*, *Bacteroides* and *Blautia* in the control group. The relative abundances of the phyla *Bacteroidetes* and *Firmicutes* and the corresponding *Firmicutes/Bacteroidetes* ratios are illustrated in **Figure 2B**, indicating that the differences were not statistically significant.

The Wilcoxon rank-sum test was performed on the top 10 genera (**Figure 2C**). *Faecalibacterium*, *Paraprevotella*, and *Prevotella* were significantly enriched in the hypothyroidism group, and *Blautia* was enriched in the control group. LEfSe analysis revealed that at the genus level, the abundances of *Faecalibacterium*, *Paraprevotella*, and *Prevotella* were strikingly increased in the hypothyroidism group, whereas those of *Anaerococcus*, *Blautia*, *Corynebacterium*, *Eggerthella, etc.* were significantly increased in the control group(**Figure 2D**).

### 3.3 Microbial compositions at the species level determined by metagenomic sequencing

**Figure 3:**
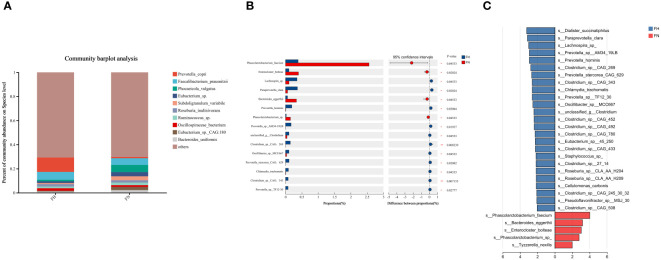
Taxonomic composition analysis by metagenomics sequencing. **(A)** The gut microbiome composition diagrams of the top 20 major species in all samples. **(B)** The top 15 species were selected for comparison. **(C)** LEfSe analysis in two groups, species with P < 0.05 and LDA scores > 2 were displayed. groupEH, the hypothyroidism group; groupEN, the control group. *P<0.05, **P<0.01.

At the species level, *Prevotella_copri*, *Faecalibacterium_prausnitzii*, *Phocaeicola_vulgatus* and *Eubacterium_sp.* were the most abundant in the two groups (**Figure 3A**). The Wilcoxon rank-sum test showed that *Paraprevotella_clara*, *Prevotella_hominis*, *Prevotella_sp_AM34_19LB*, *Prevotella_sp_TF12_30*, and *Prevotella_stercorea_CAG_629* were enriched in the hypothyroidism group, and *Phascolarctobacterium_faecium* was enriched in the control group (**Figure 3B**). LEfSe analysis revealed that the hypothyroidism group had greater relative abundances of *Prevotella_hominis*, *Prevotella_sp_AM34_19LB*, *Prevotel-la_sp_TF12_30*, *Prevotella_stercorea_CAG_629*, and *Paraprevotella_clara* (**Figure 3C**).

### 3.4 Microbial functions determined by metagenomic sequencing

**Figure 4:**
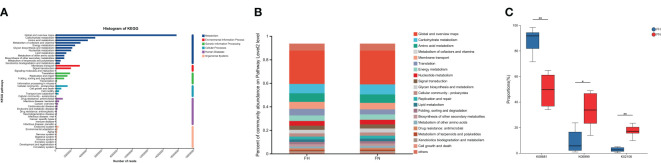
Functional analysis by metagenomics sequencing. **(A)** Genes related to KEGG pathways. Each branch represents a KEGG pathway on level 2, and different colors represent different KEGG level 1 functions. **(B)** Functional composition at Level 2 in two groups. **(C)** Boxplot of different function modules in two groups. FH, the hypothyroid group; FN, the control group. *P<0.05, **P<0.01.

Microbial KOs were identified in gut metagenomes. As shown, the metabolism was active, and dominated by global and overview maps, carbohydrate metabolism, amino acid metabolism, and metabolism of cofactors and vitamins (**Figures 4A, B**). Differences in the number of KOs between the two groups were calculated by the rank-sum test. Different functions were initially screened according to “P < 0.05”, and then, through a literature review, relevant functions were selected and displayed visually in a box plot. The hypothyroidism group had higher K08681 (pyridoxal 5’-phosphate synthase pdxT subunit [EC: 4.3.3.6]) and lower K02106 (short-chain fatty acid transporter) and K06999 (phospholipase/carboxylesterase) (**Figure 4C**).

### 3.5 Comparison of serum inflammatory factors

Serum inflammation-related factors were compared between the two groups. The serum CRP, IL-2, IL-4, IL-10 and TNF-α levels in the hypothyroidism group were greater than those in the control group (P < 0.05). There was no statistically significant difference in IL-6 between the two groups (**Table 2**).

**Table 2:** Comparison of serum inflammatory factors between the two groups.

Parameter	Hypothyroidism (n=30)	Control (n=30)	P value
CRP, mg/L#	3.86 (1.72, 6.12)	1.48 (0.67, 2.15)	**<0.001**
IL-2, pg/Ml#	0.50 (0.08, 3.08)	0.00 (0.00, 0.76)	**0.005**
IL-4, pg/mL#	2.16 (0.45, 8.32)	0.23 (0.00, 1.96)	**0.001**
IL-6, pg/mL#	2.17 (1.05, 6.67)	2.19 (1.20, 4.29)	0.953
IL-10, pg/mL#	1.30 (0.85, 5.32)	0.79 (0.28, 1.63)	**0.018**
TNF-α, pg/mL#	1.03 (0.44, 4.70)	0.53 (0.00, 1.19)	**0.017**

P<0.05 was significant. #Data are expressed as median (P25, P75). CRP, C-reaction protein; IL-2, 4, 6, 10, Interleukin-2, 4, 6, 10; and TNF-α, tumor necrosis factor α.Boldface indicates statistical significance.

### 3.6 Correlation analysis

**Figure 5:**
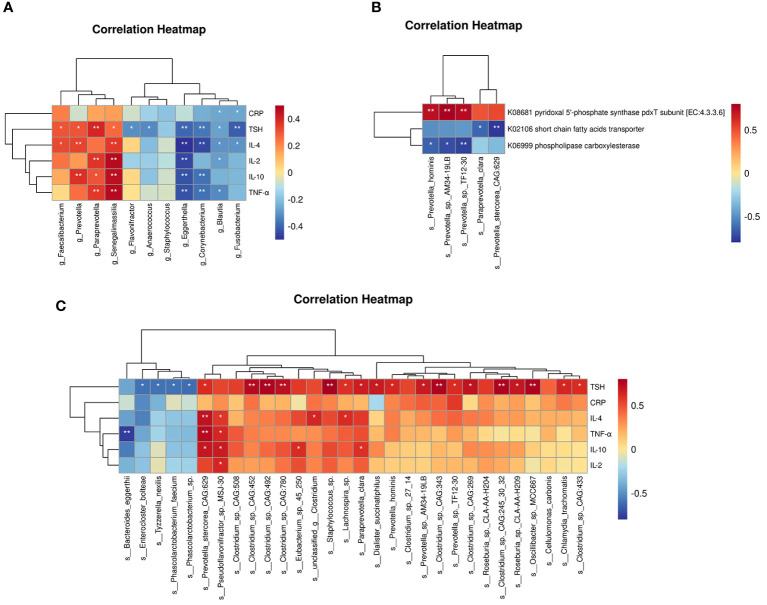
Correlation analysis of gut microbiome, serologic clinical indicators and functional modules in two groups. **(A)** Correlation analysis between different genera detected by 16S rRNA sequencing and clinical indicators. **(B)** Correlation analysis between different species detected by metagenomics sequencing and functional modules. **(C)** Correlation analysis between differential species detected by metagenomics sequencing and clinical indicators. Positive correlations are indicated in red text and negative correlations are indicated in blue text. *P < 0.05, **P < 0.01.

At the genus level (**Figure 5A**), TSH was positively correlated with *Faecalibacterium*, *Paraprevotella*, *Prevotella*, etc., and negatively correlated with *Blautia. Prevotella* was positively correlated with IL-4 and IL-10, *Paraprevotella* was positively correlated with IL-2, IL-10 and TNF-α, and *Blautia* was negatively correlated with CRP, IL-2, IL-4 and TNF-α. At the species level (**Figure 5C**), *Prevotella_stercorea_CAG_629* was positively correlated with IL-4, IL-10, and TNF-α. *Paraprevotella_clara* was positively correlated with IL-10. Correlation analysis between different microbial species and functions revealed that these three functions were associated with different species of the *Prevotella* genus (**Figure 5B**).

## 4 Discussion

Hypothyroidism is a common metabolic disease of gestation. Normal thyroid hormone levels are critical for fetal cerebral neuronal migration, synaptogenesis, and myelination (Menezes et al., 2019). Disruption of the gut microbiome can trigger immune responses and metabolic disorders in the host by damaging the integrity of the intestinal barrier, which is vital for the development and progression of thyroid diseases (Jiang et al., 2022). There is a shortage of relevant research on the application of 16S rRNA amplicon sequencing combined with shotgun metagenomics to study changes in the gut microbiome in individuals with hypothyroidism during gestation.

According to our 16S rRNA amplicon sequencing results, there was no difference in the alpha diversity of the microbiota between the two groups, while lower beta diversity was observed in the hypothyroidism group. Our study revealed a decreased abundance of *Blautia* and increased abundances of *Prevotella* and *Paraprevotella* in hypothyroid patients. Su et al. reported that the alpha diversity of the microbiota was increased in patients with primary hypothyroidism (Su et al., 2020). These results are inconsistent with our findings, which could be attributed to the fact that our subjects were pregnant women (Edwards et al., 2017). *Blautia*, a potentially beneficial bacteria, contributes to intestinal homeostasis and inflammation prevention by upregulating intestinal regulatory T cells and producing short-chain fatty acids (SCFAs) (Liu et al., 2021). *Prevotella*, an opportunistic pathogen, has been linked to autoimmune disorders, ulcerative colitis and other diseases (Sharma et al., 2022). Its cell wall component, lipopolysaccharide (LPS), can induce macrophage polarization and transform T helper (Th)1 cells into Th2 cells (Orecchioni et al., 2019; Wu et al., 2023), disrupting intestinal immune homeostasis. Furthermore, the LPS component lipid A recognizes and attaches to LPS-binding protein (LBP) in the blood, forming the LPS-LBP complex, which activates Toll-like receptor 4 (TLR-4) and mediates nuclear factor kappa-B (Di Vincenzo et al., 2023), triggering apoptosis and harming thyroid cells. Our previous studies also revealed a decreased abundance of *Blautia* and an increased abundance of *Prevotella* in the hypothyroidism group (Cai et al., 2021; Wu et al., 2023). Therefore, we suspected that the decrease in beneficial bacteria and increase in opportunistic pathogenic microbes in hypothyroid patients during early pregnancy could lead to inflammation in the intestinal tract, accelerate the development of “leaky gut”, stimulate the thyroid inflammatory immune system response, and play a role in the occurrence of hypothyroidism.

The metagenomic results revealed that, at the species level, *Paraprevotella_clara* from the *Paraprevotella* genus, *Prevotella stercorea CAG 629*, *Prevotella_hominis*, *Prevotella sp AM34_19LB*, and *Prevotella sp TF12_30* derived from the *Prevotella* genus were enriched in the hypothyroidism group, which was consistent with the 16S rRNA sequencing analysis. *Paraprevotella* currently contains only two species, and there is a lack of research on its potential role in human-health (Morotomi et al., 2009). *Prevotella* species are important sources of succinate (Hayashi et al., 2007). Succinate binds to intestinal epithelial succinate receptor 1 (SUCNR1), activating the hypoxia inducible factor 1α pathway, stimulating macrophage activation, amplifying proinflammatory effects (Fremder et al., 2021; Yan et al., 2022), and weakening the intestinal barrier. Additionally, the intracellular accumulation of succinate could reverse electron transfer and increase electron leakage in mitochondria, leading to the generation of mitochondrial reactive oxygen species (Erlich et al., 2022), and the induction of mitochondrial and endoplasmic reticulum stress (Morshed and Davies, 2020), which could contribute to thyroid cell apoptosis. We speculated that this effect might be involved in the pathogenesis of hypothyroidism during pregnancy.

According to our results, hypothyroid patients during pregnancy had increased levels of pyridoxal 5’-phosphate synthase pdxT subunit [EC: 4.3.3.6] modules and decreased levels of SCFA transporter modules and phospholipase/carboxylesterase modules. Species of *Prevotella* were significantly related to these three functional modules. We speculated that the following mechanisms could be linked to hypothyroidism: (1) Elevating pdxT could lead to increased glutamine hydrolysis and increased glutamate production (Itagaki et al., 2013). Glutamine promotes the production of intestinal secretory immunoglobulin A (SIgA) and inhibits bacterial translocation (Li et al., 2023). Its reduction could induce the upregulation of the phosphatidylinositol 3-kinase/protein kinase B (PI3K/AKT) pathway, affect the amount of the tight junction protein claudin-1, and promote intestinal permeability (Li et al., 2021). Some studies have shown glutamate-glutamine cycle abnormalities in the hippocampus of hypothyroid rats, and glutamate accumulation excessively activates neuronal cells, leading to injury or even death (Cattani et al., 2013; Domingues et al., 2018). This could explain why the hypothyroid offspring of pregnant women have a greater risk of neurological disorders. (2) SCFAs are energy substances for intestinal cells. Decreases in its transport into cells may reduce the expression of the tight junction protein claudin-1 and weaken intestinal barrier function (Xu et al., 2023). In addition, a reduction in SCFAs impairs the inhibitory effect of LPS on *Prevotella*-induced cytokine production by dendritic cells and enhances intestinal inflammation (Nastasi et al., 2015). (3) A decrease in phospholipase activity could affect the TSH signaling pathway, inhibiting thyroid hormone synthesis. Kimura et al. reported that TSH promoted the generation of hydrogen peroxide and catalyzed the oxidation and transport of iodide in FRTL-5 thyroid cells through the phospholipase C/calcium ion cascade, which assisted in thyroid hormone synthesis (Kimura et al., 1995). As suggested by the aforementioned findings, alterations to the gut microbiome could influence metabolic functional modules, increase intestinal barrier permeability, and participate in the occurrence and development of hypothyroidism during pregnancy.

We found that the CRP, IL-2, IL-4, IL-10, and TNF-α levels were greater in the hypothyroidism group than in the control group. These results indicated that women with hypothyroidism during pregnancy suffer an inflammatory response. Several investigations have shown that the serum CRP level was elevated in hypothyroid patients according to a previous study (Zhou et al., 2020; Tang et al., 2021), which is consistent with our findings. CRP interacts with phosphatidylcholine in a calcium-dependent manner in bacterial LPS, activating the complement system and enhancing macrophage phagocytosis (Volanakis, 2001). This damage to the intestinal barrier leads to a “leaky gut” and promotes intestinal bacterial LPS to enter the body’s blood circulation (Itagaki et al., 2013). Then, LPS, a pathogen-associated molecular pattern, stimulates thyroid follicular cells to express TLR-4, induces the production of regulatory T cells (Tregs) (Park et al., 2015), promotes thyroid autoimmune inflammation, and disrupts normal of thyroid function.

Our study showed that the abundance of *Prevotella* correlated positively with TSH, IL-4, and IL-10; the abundance of *Paraprevotella* correlated positively with TSH, IL-2, IL-10, and TNF-α, and the abundance of *Blautia* correlated negatively with TSH, IL-2, IL-4, and TNF-α. This indicates that women with hypothyroidism in early pregnancy have a chronic inflammatory response and that the gut microbiome may affect the thyroid autoimmune response. (1) As a potentially beneficial bacterium, lower levels of *Blautia* might result in a reduction in SCFA butyrate, a decrease in the inhibitory effect on inflammation and oxidative stress, and a promotion of intestinal inflammation (Gasaly et al., 2021). (2) LPS, a component of *Prevotella* and *Paraprevotella* cell walls, can stimulate monocyte macrophages to secrete TNF-α via the mitogen-activated protein kinase p38 signaling pathway, leading to proinflammatory damage (Dai et al., 2020). Furthermore, LPS can trigger thyroid autoimmune inflammation by encouraging TSH-stimulated thyroglobulin and sodium/iodine symporter production, leading to thyroid cell damage and hypothyroidism (Tomasello et al., 2015).

In summary, our research revealed that women with hypothyroidism in early pregnancy exhibit microbiota dysbiosis, characterized by a significant enrichment of the *Prevotella* genus. This enrichment may contribute to the onset and progression of hypothyroidism by altering the expression of glutamate, SCFAs and phospholipase. By utilizing 16S rRNA amplicon sequencing in combination with shotgun metagenomics, our study expanded the exploration of the composition and function of the gut microbiome of women with hypothyroidism in early pregnancy. However, there are still some limitations in this study. First, the number of samples in this study was small, and the enrolled individuals had certain geographic characteristics that may be influenced by their dietary habits, leading to a deviation in the results. Second, our study subjects were all pregnant women in the first trimester, and we were not directed at the dynamic changes in the gut microbiome that occur during gestation. It is necessary to conduct a longitudinal study covering the entire gestation period. In addition, an enlarged group size and multiomics experimental methods will be needed to further reveal the mechanism of hypothyroidism during pregnancy.

## Data Availability

The datasets presented in this study can be found in online repositories. The names of the repository/repositories and accession number(s) can be found below: https://www.ncbi.nlm.nih.gov/, sra/PRJNA1043851.
