# Supplementary material for: Gut microbiome characteristics of women with hypothyroidism during early pregnancy detected by 16S rRNA amplicon sequencing and shotgun metagenomic
Source: Front Cell Infect Microbiol. 2024 Aug 9;14:1369192. doi: 10.3389/fcimb.2024.1369192 (PMC11341541; doi:10.3389/fcimb.2024.1369192)
Supplement: Supplementary file 1 [file Table_1.doc]

Supplementary Table 1. Clinical characteristics of the participants in Shotgun metagenomics

| Parameter | Hypothyroidism (n=6) | Control (n=6) | P value |
| --- | --- | --- | --- |
| Maternal age, yr* | 31.17±2.40 | 29.83±2.32 | 0.351 |
| BMI, kg/m^2^* | 23.10±1.89 | 23.73±5.05 | 0.782 |
| Gestational age, weeks* | 8.29±2.16 | 10.86±2.57 | 0.091 |
| TSH, mIU/L* | 6.27±1.35 | 1.51±0.50 | **0.000** |
| FT4, mIU/L* | 14.52±2.04 | 16.45±1.83 | 0.115 |
| TgAb, n (%) | 0(0) | 0(0) | 1.000 |
| TPOAb, n (%) | 0(0) | 0(0) | 1.000 |
| TG, mmol/L# | 3.84(3.38,4.34) | 4.40(3.69,6.00) | 0.200 |
| TC, mmol/L# | 1.02(0.85,1.42) | 1.40(0.87,2.01) | 0.378 |
| LDL-C, mmol/L# | 1.59(1.25,1.97) | 1.84(1.29,2.18) | 0.522 |
| HDL-C, mmol/L# | 2.59(1.94,2.82) | 2.84(2.31,3.76) | 0.262 |
| GLU, g/L* | 123.33±8.45 | 128.00±8.53 | 0.364 |
| HGB, g/L* | 4.96±0.72 | 4.78±0.09 | 0.577 |

P<0.05 was significant. *Data are expressed as means ± standard deviation. #Data are expressed as median (P25, P75). BMI, body mass index; TSH, thyroid stimulating hormone; FT4, serum free T4; TgAb, anti-thyroglobulin antibodies; ,TPOAb, thyroid peroxidase antibody; TC, serum total cholesterol; TG, triglycerides; LDL-C, low-density lipoprotein cholesterol; HDL-C, high-density lipoprotein cholesterol; GLU, fasting blood glucose; and HGB, hemoglobin.
